# Axonopathy precedes cell death in ocular damage mediated by blast exposure

**DOI:** 10.1038/s41598-021-90412-2

**Published:** 2021-06-03

**Authors:** Nickolas A. Boehme, Adam Hedberg-Buenz, Nicole Tatro, Michael Bielecki, William C. Castonguay, Todd E. Scheetz, Michael G. Anderson, Laura M. Dutca

**Affiliations:** 1 Center for the Prevention and Treatment of Visual Loss, Iowa City VA Healthcare System, 601 Hwy 6 W (151), Iowa City, IA 52772 USA; 2 Department of Ophthalmology and Visual Science, The University of Iowa, Iowa City, IA USA; 3 Department of Molecular Physiology and Biophysics, The University of Iowa, Iowa City, IA USA; 4 Institute for Vision Research, The University of Iowa, Iowa City, IA USA

**Keywords:** Visual system, Neuroscience

## Abstract

Traumatic brain injuries (TBI) of varied types are common across all populations and can cause visual problems. For military personnel in combat settings, injuries from blast exposures (bTBI) are prevalent and arise from a myriad of different situations. To model these diverse conditions, we are one of several groups modeling bTBI using mice in varying ways. Here, we report a refined analysis of retinal ganglion cell (RGC) damage in male C57BL/6J mice exposed to a blast-wave in an enclosed chamber. Ganglion cell layer thickness, RGC density (BRN3A and RBPMS immunoreactivity), cellular density of ganglion cell layer (hematoxylin and eosin staining), and axon numbers (paraphenylenediamine staining) were quantified at timepoints ranging from 1 to 17-weeks. RNA sequencing was performed at 1-week and 5-weeks post-injury. Earliest indices of damage, evident by 1-week post-injury, are a loss of RGC marker expression, damage to RGC axons, and increase in glial markers expression. Blast exposure caused a loss of RGC somas and axons—with greatest loss occurring by 5-weeks post-injury. While indices of glial involvement are prominent early, they quickly subside as RGCs are lost. The finding that axonopathy precedes soma loss resembles pathology observed in mouse models of glaucoma, suggesting similar mechanisms.

## Introduction

Traumatic brain injury (TBI) is common and can have many consequences, including impacts on the visual system. The number of people in the civilian population who suffer from TBI is not precisely known, but it is clearly large. In the United States, more than 2 million TBI-related emergency department visits, hospitalizations, and deaths are recorded each year^1^. In addition, TBIs are common in military personnel^2^. Although most TBIs (~ 80%) are classified as “mild”^2^, they are often associated with cognitive, behavioral, emotional, and sensorimotor problems^3–5^. In addition, vision complaints have been reported in up to 79% of TBIs^6–9^. Currently, the molecular and cellular mechanisms that contribute to TBI-associated visual loss are not well understood, and whether responses differ according to the mechanism of injury is unknown^10,11^.

One approach for studying the molecular events that underlie the changes in ocular structure that are associated with TBI is using animal models. Like several other groups^12–18^, we have been particularly interested in using mice to study blast-induced TBI (bTBI). The primary model we have utilized for this is the exposure of mice to a 20 pounds per square inch (PSI) blast-wave in an enclosed chamber. This model mimics aspects of mild blast injury through the exposure to a pressure wave, but does not involve globe rupture or exposure to heat or shrapnel^19–24^. Previous studies using this model revealed consequences such as axonal degeneration, abnormal synaptic activity, defective learning and memory, and impaired motor coordination^25,26^. The ocular damage resulting from this treatment affects primarily retinal ganglion cells (RGCs), as apparent from a thinning of the retinal layers that comprise the ganglion cell complex (GCC)^19^, a loss of RGC somas^20,23^, and loss of axons from the optic nerve^21^. Although RGC damage has been observed consistently in several studies, a systematic analysis of the retina and optic nerve at the same time-point to define a timeline of the events has not been performed and few attempts have been made to start defining the earliest stages of damage in this model.

Here we report the effects of blast exposure on the retina and optic nerve. To refine the timeline of the events involved in RGC damage, we took several approaches. We used spectral domain optical coherence tomography (SD-OCT) images taken at timepoints ranging from 1 to 17 weeks after injury and assessed the effect on thickness of the GCC. We complemented this with quantification of: RGC density based upon quantification of BRN3A immunoreactivity (BRN3A^+^), a transcription factor also known as POU4F1 which labels RGCs^27^, in retinal whole-mounts; density of the all the cells of the ganglion cell layer based on hematoxylin and eosin (H&E) staining in retinal whole-mounts; and myelinated axons in the RGC layer based on manual quantification in optic nerves stained with paraphenylenediamine (PPD), a dye that stains myelin. To identify early molecular changes, the retinal transcriptomes of the retinas of injured and sham mice were analyzed by RNA sequencing (RNA-Seq) at 1 week and 5 weeks after blast injury and compared. Collectively, the results of these experiments suggest that the earliest indices of damage are a loss of BRN3A expression, damage of RGC axons, and multiple molecular changes indicative of a role for microglia and astrocytes in the damage. These changes are evident by 1-week post-injury and followed by a loss of 25–30% of RGC somas and axons—with the greatest loss occurring by 5 weeks post-injury. While the indices of the glial contribution to the damage appear in the early stage disease, they quickly subside as RGCs are lost.

## Results

### Early structural events of bTBI are discernable by 1-week post injury

**Figure 1:**
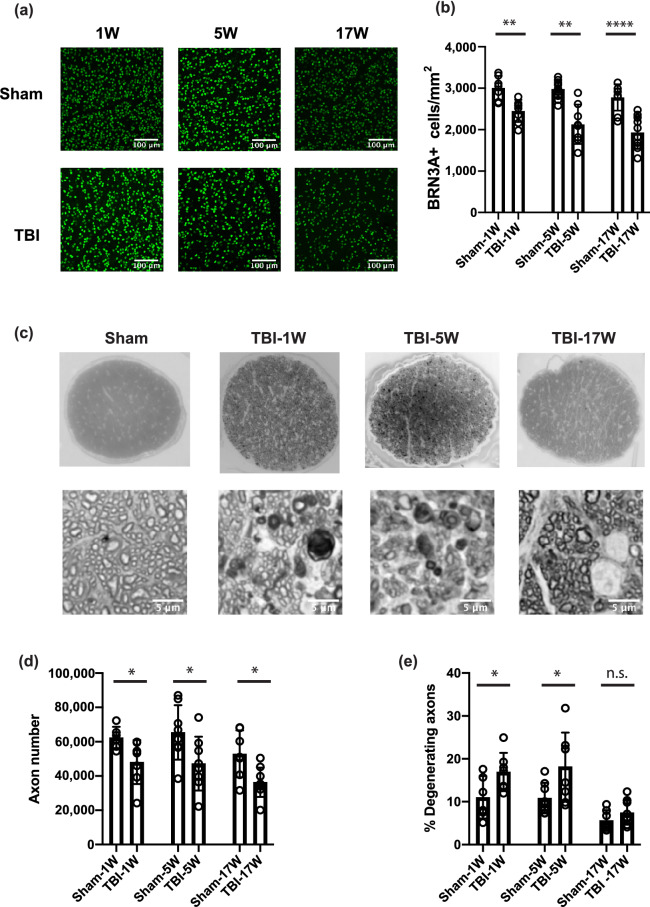
Blast-mediated traumatic brain injury (bTBI) was associated with loss of BRN3A^+^ retinal ganglion cells (RGCs) and axons. (**a**) Representative images from the mid-central area of whole-mount retinas stained with anti-BRN3A antibody (green) at the indicated time-point. Mice were sham treated or subjected to bTBI. (**b**) Quantification of BRN3A^+^ RGCs at 1 week (1 W), 5 weeks (5 W) and 17 weeks (17 W) after injury. (**c**) Representative images of paraphenylenediamine (PPD)-stained optic nerves from sham mice and bTBI mice at 1 W, 5 W, and 17 W after injury, at low (upper row) and high (lower row) magnification. (**d**) Quantification of axons in PPD-stained optic nerves at 1 W, 5 W, and 17 W after injury. The optic nerves examined were from the retinas in which the BRN3A^+^ cells were quantified. (**e**) Percent of degenerating axons in PPD-stained optic nerves at 1 W, 5 W and 17 W after injury. Bars represent mean ± SD and each data point represents a biological sample; unpaired two tailed *t*-test with Welch’s correction: *p < 0.05, **p < 0.01, **** p < 0.0001, *n.s.* not significant. The images were processed in Fiji^111^, the graphs were generated in Prism 8 software and the figure was assembled in Adobe Illustrator CC 2014.

**Figure 2:**
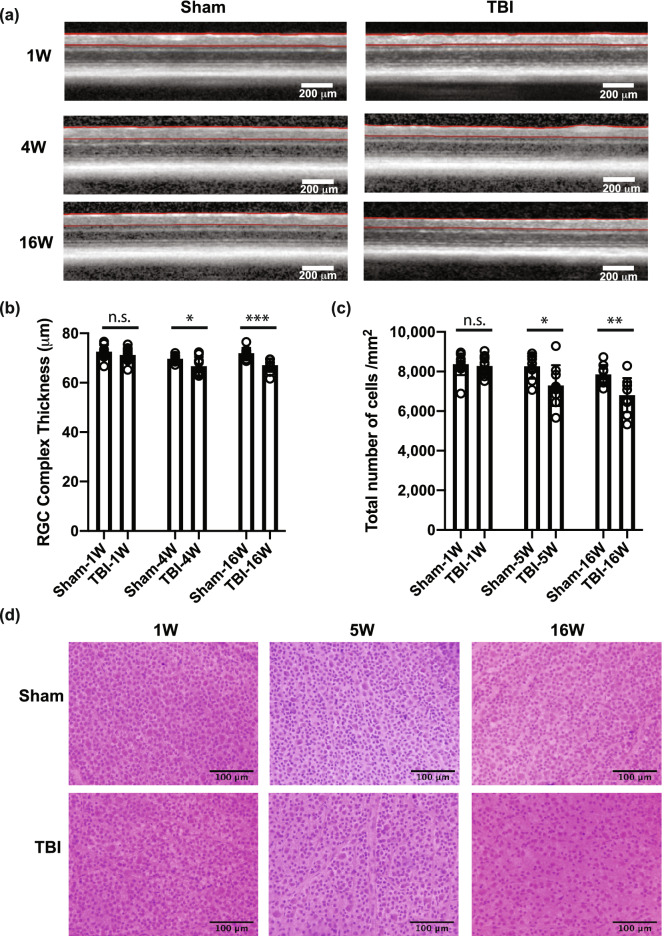
At 5 weeks post bTBI, cell loss from the ganglion cell layer was significant. (**a**) Images of the retina acquired by spectral domain optical coherence tomography (SD-OCT) at the indicated time points, with the ganglion cell complex (GCC) highlighted by red lines. (**b**) Representative images from the mid-central area of whole-mount retinas from sham and injured mice at the indicated time points, stained with hematoxylin and eosin (H&E). (**c**) Thickness of the GCC in vivo, as determined by SD-OCT at 1 week (1 W), 4 weeks (4 W), and 16 weeks (16 W) after bTBI. (**d**) Density of all cells in ganglion cell layer was quantified in whole-mount retinas stained with H&E at 1 W, 5 W, and 16 W after injury. Bars represent mean ± SD and each data point represents a biological sample; unpaired two tailed *t*-test analysis with Welch’s correction: *p < 0.05, **p < 0.01, ***p < 0.001, *n.s*. not significant. The images were processed in Fiji^111^, the graphs were generated in Prism 8 software and the figure was assembled in Adobe Illustrator CC 2014.

To define the timeframe of the early events that contribute to RGC damage as ocular damage induced by blast exposure develops, we performed SD-OCT (for assessment of thickness of the GCC) and histochemical and immunohistochemical staining of the retinas of mice (to assess cell degeneration and loss), at 1, 4–5, and 16–17 weeks following bTBI. Indices of RGC damage were first detected in the 1-week cohort (Fig. 1; Supplementary Tables S1 and S2, and Supplementary Fig. S1 online). These include: a statistically significant reduction in the density of BRN3A^+^ immunoreactive nuclei (2451 ± 267.2 cells/mm^2^ vs. 3008 ± 288.2 cells/mm^2^, *p* = 0.021) (Fig. 1a,b); a reduction in the total number of myelinated axons in the optic nerve (48,117 ± 12,854 axons vs. 62,368 ± 6329 axons, *p* = 0.0056) (Fig. 1c,d); and an increase in the number of degenerating myelinated axons (16.94 ± 4.45% vs 11.03 ± 4.95%, *p* = 0.0116) (Fig. 1c,e). Loss of BRN3A^+^ cells was significant in all areas (central, mid-central, peripheral) of the retina (Supplementary Fig. S1 online). At this time, no significant changes in GCC thickness (71.20 ± 2.54 μm vs 72.58 ± 2.65 μm, *p* = 0.1665) (Fig. 2a,b) or the total density of cells in the ganglion cell layer (8281 ± 451 cells/mm^2^ vs. 8367 ± 547 cells/mm^2^, *p* = 0.6733) were detected (Fig. 2c,d). At 5 weeks post bTBI RGC damage had worsened (Fig. 1), as evident from further: decreases in density of BRN3A^+^ cells (2130 ± 474 cells/mm^2^ vs. 2981 ± 222.1 cells/mm^2^, *p* = 0.001); decreases in the total number of myelinated axons (47,256 ± 15,731 vs. 65,433 ± 15,860, *p* = 0.0373); and increases in the number of degenerating myelinated axons (18.18% ± 7.97% vs. 10.85 ± 3.57%, *p* = 0.0390). In addition, GCC thickness decreased by 4 weeks post-bTBI (66.70 ± 3.71 μm vs. 69.68 ± 1.43 μm, *p* = 0.0367), and total cellularity in the RGC layer decreased by 5 weeks post-TBI (7294 ± 1030 cells/mm^2^ vs 8263 ± 622 cells/mm^2^, *p* = 0.0309) (Fig. 2). At 16–17 weeks post-bTBI, the phenotypes were similar to those observed at 4–5 weeks (Figs. 1, 2; Supplementary Tables S1 and S2 online), with significant differences detected between the bTBI and sham groups by all analyses.

**Figure 3:**
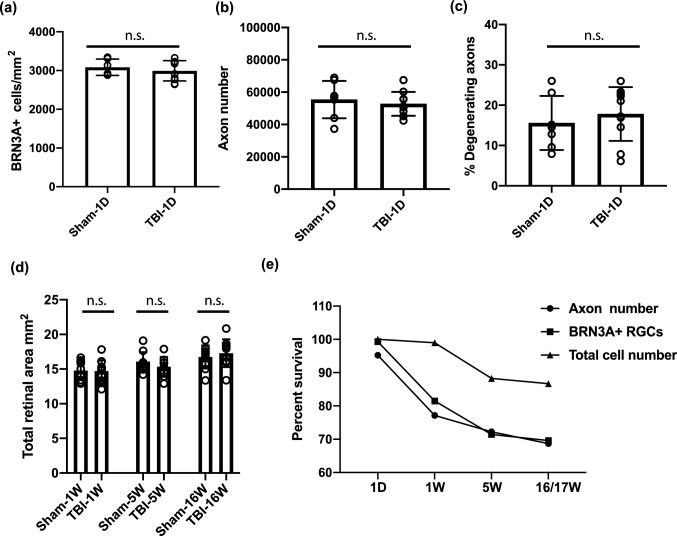
At 1 day after injury, no significant changes were detected in the retina or optic nerve. (**a**) Quantification of BRN3A^+^ RGCs at 1 day (1D) post blast exposure. (**b**) Quantification of axons 1D after injury. (**c**) Percent of degenerating axons in the optic nerves 1D after injury. (**d**) Total retinal area of hematoxylin and eosin (H&E) stained whole-mount retinas at 1 week (1 W), 5 weeks (5 W), and 16 weeks (16 W) after injury. (**e**) Percent survival of BRN3A^+^ RGCs, PPD stained axons, and total number of H&E stained cells in the ganglion cell layer. For the total cell count in the ganglion cell layer at the 1D time-point, 100% survival was assumed rather than determined experimentally. Bars represent mean ± SD and each data point represents a biological sample; unpaired two tailed *t*-test analysis with Welch’s correction, *n.s.* not significant. The graphs were generated in Prism 8 software and the figure was assembled in Adobe Illustrator CC 2014.

Additional experiments were conducted to facilitate interpretation of these findings. First, to refine the time window in which RGC damage occurred, we analyzed a cohort of mice with a subset of the above-described assays at 1-day post-injury (Fig. 3a–c; Supplementary Table S1 online). No statistically significant differences were observed between the bTBI and sham groups with respect to the density of BRN3A^+^ RGCs (3063 ± 272.2 cells/mm^2^ vs. 3084 ± 210.2 cells/mm^2^, *p* = 0.4546), the number of myelinated axons (52,750 ± 7378 axons vs 55,400 ± 11,532 axons, *p* = 0.604), or the percentage of degenerating myelinated axons (17.81% ± 6.67 vs 15.59 ± 6.69%). Second, as an additional control, we measured the areas of the retinal whole-mounts (Fig. 3d; Supplementary Table S2 online). Because retinal area increases by up to ~ 20% in some mouse models of glaucoma^28^, and might also change in bTBI, this is an important test to discern whether changes in density are related to cell number or area. However, no differences in the retinal area were detected (Fig. 3d; Supplementary Table S2 online). Third, to confirm our methodology and the loss of RGCs observed by BRN3A staining, an independent cohort of sham and bTBI mice was analyzed at the 1-week timepoint using two RGC markers (RBPMS and BRN3A, instead of only BRN3A). Manual counting of peripheral and mid-central fields (instead of automated, see Experimental Methods) was performed to determine the density of RBPMS ^+^ and BRN3A^+^ cells. Among the sham treated mice, RBPMS labeled a slightly larger population of cells compared to BRN3A (average 2830 vs 2577 cells/mm^2^; 8.9% more cells). However, both markers showed equivalent decreases in labeled cells at 1-week following TBI (RBPMS, − 275 cells/mm^2^; BRN3A, − 274 cells/mm^2^; Supplementary Fig. S2 online).

To assess congruency between our cellular assays, we compared outcomes as the percentage of damage relative to that in the respective sham controls. The reduction of BRN3A^+^ density in bTBI-treated vs sham mice was 18.52% at 1 week, 28.55% at 5 weeks, and 30.39% at 17 weeks (Fig. 3e). The reduction of total cellular density in the ganglion cell layer was 1.0% at 1 week, 11.7% at 5 weeks, and 13.3% at 16 weeks. Thus, the percent loss of BRN3A^+^ cells was consistently greater than the loss of total cellularity in the RGC layer. With an assumption that ~ 50% of the cells in the ganglion cell layer are RGCs (based on reports of 41–61%, depending on the counting method^29–32^), the total cellularity data is highly congruent with the BRN3A data at the 5- and 16-week timepoints (representing losses of 23.4% at 5 weeks and of 26.6% at 16 weeks for the total cell number), suggesting that the cell loss is purely due to RGC loss and does not impact displaced amacrine cells. However, the data for the 1-week timepoint is incongruent (a loss of 2% of the total cell number) with the later timepoints, indicating that a loss of BRN3A^+^ expression (Fig. 1a,b) occurred prior to the loss of actual cells (Fig. 2c,d). There was also temporal incongruency in a consideration of RGC axons vs. soma; axon loss (Fig. 1c,d) and axon damage (Fig. 1c,e) occurred prior to cellular loss (Fig. 2c,d). These comparisons indicate that early-stage retinal damage in this model of bTBI manifests initially as axonopathy. At molecular level the early-stage retinal damage presents as loss of RGC markers (BRN3A and RBPMS).

### The retinal transcriptome changes after bTBI

**Figure 4:**
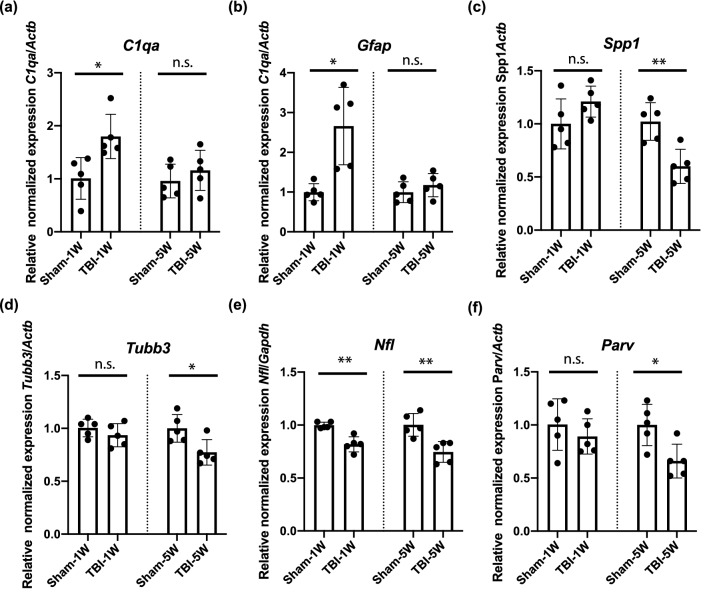
bTBI-associated changes in transcript levels observed by RNA sequencing confirmed by quantitative reverse transcription PCR of a subset of affected transcripts. Relative expression of (**a**) *C1qa*, (**b**) *Gfap*, (**c**) *Spp1*, (**d**) *Tubb3*, (**e**) *Nfl,* and (**f**) *Parv* normalized to the level of *Actb* or *Gapdh*, as indicated, at 1 week (1 W) or 5 weeks (5 W) and also normalized to the mean of the sham for that time-point. Bars represent mean ± SD and each data point represents a biological sample (n = 5 per group); unpaired two tailed *t*-test analysis with Welch’s correction: *p < 0.05, **p < 0.01 and *n.s.* not significant. The graphs were generated in Prism 8 software and the figure was assembled in Adobe Illustrator CC 2014.

To further identify the early events that occur in the retina following bTBI, we analyzed the retinal transcriptome of mice exposed to blast by RNA-Seq. At 1-week post-bTBI, 335 genes were differentially expressed in the bTBI vs. sham group (Table 1, full data available in Supplementary Table S3 online), with 197 genes upregulated and 138 downregulated; at 5 weeks post-bTBI, 175 transcripts were differentially expressed (Table 1, full data available in Supplementary Table S4 online), with 4 upregulated and 171 downregulated (Table 1). The most highly upregulated and downregulated transcripts in the retinas of bTBI versus sham mice at 1 and 5 weeks after injury, are presented in Tables 2 and 3, respectively. To test whether these results were an artifact of the RNA-Seq approach (Table 2), 6 transcripts corresponding to RGC markers *(Nefl*, *Parv*, *Spp1* and *Tubb3*) and associated with an immune response (*Gfap*, *C1qa* and *Spp1*) were independently tested by quantitative reverse transcription PCR (RT-qPCR) (Fig. 4a–f). Among these, 5 exhibited statistically significant changes in the expected direction (Fig. 4a–e), and changes in the sixth (*Parv*) were consistent in direction but the difference was not statistically significant (Fig. 4f). At the two timepoints, 101 of the same transcripts were differentially expressed (Supplementary Table S5 online), including all 4 of those that were upregulated at the 5-week timepoint (*Ecel1*, *Lad1*, *Pros1* and *Impg2*; Table 3).

**Table 1:** Summary of RNA sequencing results of retinal transcripts from bTBI- versus sham-treated mice.

Gene expression in TBI versus Sham	1 week	5 weeks
Differentially expressed	335	175
Increased expression	197	4
Decreased expression	138	171
Log_2_FC range	− 1.65 to 4.79	− 3.06 to 1.42
FC range	0.32 to 27.7	0.12 to 2.67
FDR cut-off	< 0.005	< 0.005
Shared change in expression	101	

*FC* fold change

*FDR* false discovery rate.

**Table 2:** Most highly up- or downregulated retinal transcripts in bTBI- versus sham-treated mice at 1-week post injury detected by RNA sequencing.

Symbol	Transcript	Fold change	*p*-value	FDR
**Upregulated**				
*Mmp12*	Matrix metallopeptidase 12	27.83	2.48E−10	3.70E−06
*Ecel1*	Endothelin converting enzyme-like 1/DINE	14.60	3.11E−08	6.76E−05
*6430562O15Rik*	Long non-coding RNA	2.03	2.88E−08	6.76E−05
*Oasl2*	2′–5′ Oligoadenylate synthetase-like 2	4.23	2.91E−08	6.76E−05
*Isg15*	ISG15 ubiquitin-like modifier	3.82	3.17E−08	6.76E−05
*Gbp2*	Guanylate binding protein 2	3.80	1.89E−08	6.76E−05
*Ptgfr*	Prostaglandin F receptor	4.30	1.08E−07	0.00018
*Irf9*	Interferon regulatory factor 9	2.80	1.01E−07	0.00018
*Cd180*	CD180 antigen	6.21	1.30E−07	0.00019
*Psmb8*	Proteasome (prosome, macropain) subunit, beta type 8	2.65	1.51E−07	0.00019
**Downregulated**				
*Limk1*	LIM-domain containing, protein kinase	0.63	3.05E−08	6.76E−05
*Lynx1*	Ly6/neurotoxin 1	0.72	2.60E−07	0.0002
*Dlgap3*	DLG associated protein 3	0.74	4.81E−07	0.00026
*Tubb3*	Tubulin, beta 3 class III	0.73	5.15E−07	0.00026
*Cend1*	Cell cycle exit and neuronal differentiation 1	0.67	5.04E−07	0.00026
*Kcnip4*	Potassium voltage-gated channel interacting protein 4	0.65	6.54E−07	0.00029
*Nat8l*	*N*-Acetyltransferase 8-like	0.71	9.74E−07	0.00035
*Apba1*	Amyloid beta (A4) precursor protein binding, family A, member 1	0.76	1.05E−06	0.00035
*Nefm*	Neurofilament, medium polypeptide	0.61	1.23E−06	0.00036

*FDR* false discovery rate.

**Table 3:** Most highly up- or downregulated retinal transcripts in bTBI- vs. sham-treated mice at 5-weeks post injury detected by RNA sequencing.

Symbol	Description	Fold change	*p*-value	FDR
**Upregulated**				
*Ecel1*	Endothelin converting enzyme-like 1/dine	2.68	1.16E−06	2.10E−04
*Lad1*	Ladinin	1.78	3.48E−05	3.46E−03
*Pros1*	Protein S, alpha	1.37	3.04E−05	3.16E−03
*Impg2*	Interphotoreceptor matrix proteoglycan 2	1.10	4.93E−05	4.67E−03
**Downregulated**				
*Cplx1*	Complexin 1	0.56	2.77E−13	4.40E−09
*Nefm*	Neurofilament, medium polypeptide	0.56	2.67E−12	1.54E−08
*Vamp1*	Vesicle-associated membrane protein 1	0.70	2.90E−12	1.54E−08
*Rgs4*	Regulator of G-protein signaling 4	0.59	1.37E−11	5.46E−08
*Thy1*	Thymus cell antigen 1, theta	0.70	4.66E−11	1.39E−07
*Coro6*	Coronin 6	0.53	5.59E−11	1.39E−07
*Sncg*	Synuclein, gamma	0.59	6.12E−11	1.39E−07
*Nefl*	Neurofilament, light polypeptide	0.60	8.63E−11	1.72E−07
*Lynx1*	Ly6/Neurotoxin 1	0.77	1.11E−10	1.96E−07
*Nefh*	Neurofilament, heavy polypeptide	0.62	1.42E−10	2.26E−07

*FDR* false discovery rate.

**Figure 5:**
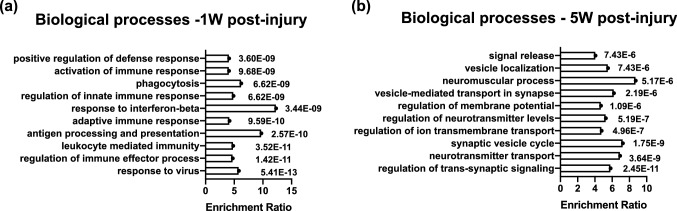
Transcriptomic analysis of the retina after exposure to blast with RNA sequencing. (**a**) Transcripts whose expression was significantly dysregulated at (**a**) 1 week (1 W) or (**b**) 5 weeks (5 W). Transcripts whose expression changed significantly (false discovery rate (FDR) < 0.005 for TBI compared to sham retinas) analyzed using WebGestalt, by performing an “Over-Representation Analysis” using the gene ontology database for “Biological Process noRedundant” analysis. The false discovery rate (FDR) for each process is indicated. The graphs were generated using WebGestalt 2019^109^ and the figure was assembled in Adobe Illustrator CC 2014.

**Figure 6:**
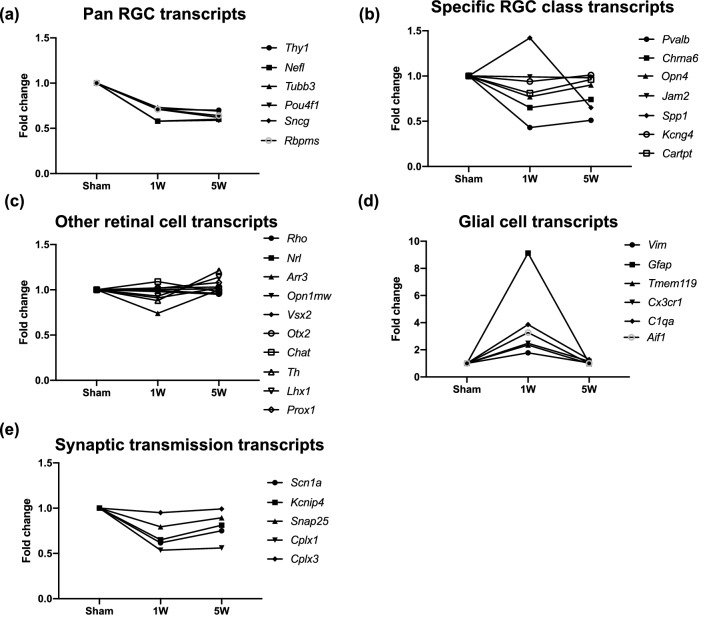
RNA sequencing of the retinal transcriptome shows differences in RGC and glial specific transcripts in bTBI vs. sham retinas. Shown is expression of markers for: (**a**) all RGCs, (**b**) classes of RGCs, (**c**) other types of retinal cells, (**d**) immune cells, and (**e**) synaptic transmission. Affected transcripts of each type are indicated for the corresponding graph. The names of the transcripts and the false discovery rates from RNA sequencing are provided in Table 4. The graphs were generated using WebGestalt 2019^109^ and the figure was assembled in Adobe Illustrator CC 2014.

Gene ontology (GO) and pathway analysis using the WebGestalt toolkit indicated that the pattern of changes in gene expression was complex, including early upregulation of microglia and macroglia-related transcripts, as well as sustained downregulation of several genes important to neuronal function (Fig. 5a,b). Transcripts with well-known links to specific pathways or cell types were additionally analyzed in a case-by-case manner. Consistent with the immunohistochemical and histologic analyses, transcripts of several genes regarded as broad markers for multiple types of RGCs were significantly down-regulated (by ~ 25–30%) by 1-week post-injury (Fig. 6a; Table 4). However, there was also evidence that some changes in RGC gene expression were specific to certain RGC sub-types (Fig. 6b; Table 4). In contrast, markers of non-RGC retinal cell types were uniformly unaltered (Fig. 6c; Table 4). Transcripts associated with several kinds of cells relevant to immune responses of the retina were significantly upregulated at 1-week, but not 5-weeks, post-injury. Examples in this category include *Vim* (Müller glia), *Gfap* and *Nes* (Müller glia and astrocytes), *Tmem119*, *Aif1* and *Cx3cr1* (microglia), and *C1qa* (microglia and Müller glia) (Fig. 6d; Table 4). In the case of transcripts encoding synaptic proteins (*Snap25*, *Cplx1*) and ion channels (*Scn1a*, *Kcnip4*), some were significantly downregulated but others were not (*Cplx3*), (Fig. 6e; Table 4).

**Table 4:** Changes in expression (fold change - FC) and false discovery rate (FDR) for transcripts related to retinal ganglion cells, retinal cells and glial cells at 1- and 5-weeks post injury.

Time-point			1 week		5 weeks	
Gene name	Name	Expressed in	logFC	FDR	logFC	FDR
*Rho*	Rhodopsin	Rods	− 2.35E−02	9.20E−01	3.84E−02	6.22E−01
*Nrl*	Neural retina leucine zipper gene	Rods	− 1.93E−02	9.06E−01	6.17E−02	3.43E−01
*Arr3*	Arrestin 3, retinal	Cones	− 4.29E−01	6.08E−03	2.14E−03	9.94E−01
*Opn1mw*	Opsin 1 medium-wave-sensitive	Cones	− 1.28E−01	3.74E−01	2.92E−02	8.87E−01
*Vsx2*	Visual system homeobox 2	Bipolar cells	3.64E−03	9.88E−01	2.15E−02	9.19E−01
*Otx2*	Orthodenticle homeobox 2	Bipolar cells	3.68E−02	7.20E−01	4.75E−02	5.10E−01
*Chat*	Choline acetyltransferase	Amacrine cells	− 1.31E−01	3.91E−01	− 2.46E−02	9.47E−01
*Th*	Tyrosine hydroxylase	Amacrine cells	− 1.81E−01	5.84E−01	2.79E−02	9.61E−01
*Lhx1*	LIM homeobox protein 1	Horizontal cells	− 1.06E−01	8.07E−01	1.86E−01	5.50E−01
*Prox1*	Prospero homeobox 1	Horizontal cells	2.53E−02	9.36E−01	1.15E−01	7.56E−02
*Vim*	Vimentin	Muller glia	8.32E−01	3.13E−03	5.37E−02	6.08E−01
*Gfap*	Glial fibrillary acidic protein	Muller glia/astrocytes	3.19E+00	2.40E−03	4.05E−01	2.32E−01
*Tmem119*	Transmembrane protein 119	Microglia	1.23E+00	1.19E−03	− 2.88E−02	9.74E−01
*Cx3cr1*	Chemokine (C-X3-C motif) receptor 1	Microglia	1.31E+00	3.51E−04	1.97E−01	5.31E−01
*Aif1*	Allograft inflammatory factor 1	Microglia	1.72E+00	3.59E−04		
*C1qa*	Complement component 1, q subcomponent, alpha polypeptide	Microglia	1.95E+00	1.20E−03	3.77E−01	1.09E−01
*Nefm*	Neurofilament, medium	RGCs	− 7.11E−01	3.64E−04	− 8.41E−01	1.54E−08
*Thy1*	Thymus cell antigen 1, theta	RGCs	− 4.94E−01	1.31E−03	− 5.14E−01	1.39E−07
*Sncg*	Synuclein, gamma	RGCs	− 7.70E−01	9.40E−04	− 7.66E−01	1.39E−07
*Nefl*	Neurofilament, light	RGCs	− 7.86E−01	1.01E−03	− 7.48E−01	1.72E−07
*Nefh*	Neurofilament, heavy	RGCs	− 9.31E−01	4.43E−04	− 6.92E−01	2.26E−07
*Rbpms*	RNA binding protein with multiple splicing	RGCs	− 5.06E−01	5.84E−03	− 6.48E−01	2.53E−07
*Tubb3*	Tubulin, beta 3 class III	RGCs	− 4.55E−01	2.62E−04	− 5.33E−01	3.65E−07
*Pvalb*	Parvalbumin	RGCs	− 1.20E+00	1.18E−03	− 9.79E−01	1.62E−06
*Pou4f1*	POU domain, class 4, transcription factor 1	RGCs	− 4.98E−01	5.63E−04	− 6.93E−01	3.52E−05
*Pou4f2*	POU domain, class 4, transcription factor 2	RGCs	− 5.17E−01	4.41E−02	− 5.55E−01	6.10E−04
*Pou4f3*	POU domain, class 4, transcription factor 3	RGCs	− 6.08E−01	5.70E−02	− 7.95E−01	2.02E−03
*Rbfox3*	RNA binding protein, fox-1 homolog (C. elegans) 3	RGCs	− 3.81E−01	4.20E−03	− 3.48E−01	1.93E−03
*Chrna6*	Cholinergic receptor, nicotinic, Alpha polypeptide 6	RGCs	− 6.14E−01	5.61E−04	− 4.41E−01	4.12E−06
*Opn4*	Opsin 4 (melanopsin)	ipRGCs	− 3.70E−01	2.35E−02	− 1.54E−01	6.21E−01
*Jam2*	Junction adhesion molecule 2	J-RGCs	− 3.92E−03	9.83E−01	− 3.29E−02	8.06E−01
*Spp1*	Osteopontin	alpha RGCs	5.11E−01	2.00E−02	− 6.05E−01	2.19E−04
*Kcng4*	Potassium voltage-gated channel, subfamily G, member 4	alpha RGCs	− 8.92E−02	5.28E−01	1.24E−02	9.60E−01
*Cartpt*	Potassium voltage-gated channel, subfamily G, member 4	ooDSGC	− 3.16E−01	1.58E−01	− 6.04E−02	7.74E−01

### Glia are activated in the retina at 1 week after bTBI

**Figure 7:**
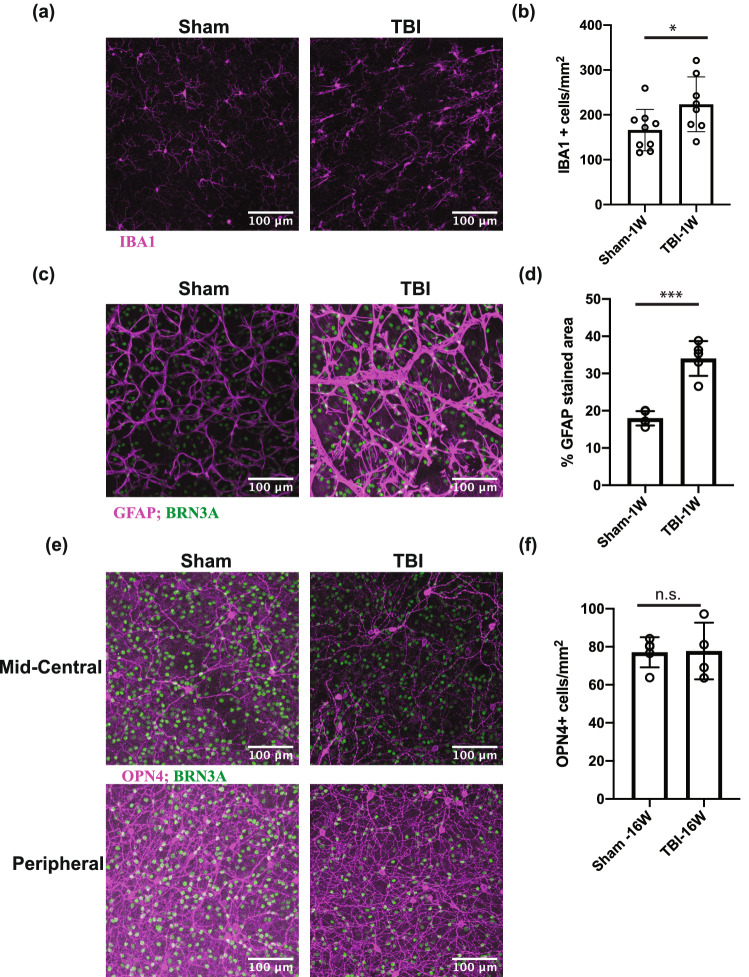
Blast-mediated injury resulted in a significant increase in the number of microglia in the retina within one week. (**a**) Images from the mid-central area of whole-mount retinas stained with anti-IBA1 antibody (pink) at 1 week (1 W) post-injury, for sham and bTBI-treated mice. (**b**) Quantification of IBA1^+^ cells at 1 W after injury. (**c**) Images from the mid-central area of whole-mount retinas stained with anti-GFAP antibody (pink) and anti-BRN3A antibody (green) at 1 W post-injury, for sham and bTBI-treated mice. (**d**) Quantification of GFAP^+^ area 1 W after injury. (**e**) Images from the mid-central and peripheral areas of whole-mount retinas stained with anti-OPN4 antibody (pink) and anti-BRN3A antibody (green) at 16 weeks (16 W) post-injury for sham and bTBI-treated mice. (**f**) Quantification of OPN4^+^ positive cells at 16 W after injury. Bars represent mean ± SD and each data point represents a biological sample; unpaired two tailed *t*-test analysis with Welch’s correction, *p < 0.05, ***p<0.001, *n.s.* not significant. The images were processed in Fiji^111^, the graphs were generated in Prism 8 software and the figure was assembled in Adobe Illustrator CC 2014.

The changes in some of the glial transcripts observed by RNA-Seq were confirmed at the protein level, in particular those for the *Aif1* transcript corresponding to the protein AIF-1/IBA1 in microglia and the *Gfap* transcript corresponding to GFAP in astrocytes and Müller glia. The microglia in whole-mount retinas were stained with anti-IBA1 (Fig. 7a,b) at 1 week after injury, and the total number of IBA1^+^ cells was significantly increased (223 ± 60.97 cells/mm^2^ vs 166 ± 46.18 cells/mm^2^, *p* = 0.0494). To assess the activation of astrocytes and Müller glia, retinal whole mounts were stained with anti-GFAP (see Fig. 7c,d). The percent area of the inner retinal layer that was stained by the GFAP antibody was significantly larger in the bTBI- vs sham-treated retinas (34.02 ± 4.66% vs 17.97 ± 1.94 cells/mm^2^, *p* = 0.0005).

### The number of OPN4^+^ retinal ganglion cells is not affected by bTBI

To further confirm the changes at transcript level, we also analyzed the changes in a protein corresponding to a transcript that did not change (*Opn4*), as a negative control. In particular, we determined the density of RGCs expressing melanopsin (OPN4), i.e., the intrinsically photosensitive RGCs (ipRGCs). Furthermore, the RGCs that express melanopsin, mostly do not express BRN3A, and are thought to play a role in photophobia, one of the common outcomes of TBI^33^. IpRGCs were quantified in whole-mount retinas at 16 weeks post-bTBI (Fig. 7e,f). The density of these cells did not differ significantly in injured vs sham mice (77.77 ± 14.90 cells/mm^2^ vs 77.10 ± 7.92 cells/mm^2^, *p* = 0.9390).

## Discussion

Collectively, our data indicate that the earliest structural damage in the blast injury model is damage to RGC axons, and molecularly, there is a loss of RGC marker expression and an increase in glial markers expression. These changes were detectable at 1-week post-bTBI. The level of the BRN3A-encoding transcript (*Pou4f1*) was also decreased at 1-week post-bTBI (to 71%), along with those of transcripts encoding pan markers of RGCs. Because the loss of BRN3A and RBPMS immunoreactivity at 1-week post-bTBI was not accompanied by a loss of total cellularity in the ganglion cell layer, this initial loss of BRN3A and RBPMS immunolabeling presumably represents a stress-related loss of the expression rather than cell death. The retinal transcriptome at 1-week post-bTBI revealed many changes associated with microglia and macroglia (Müller glia and astrocytes) and immunohistochemical analysis confirmed some of these changes. By 5-weeks post-bTBI, the results from all of our cellular assays were congruent, indicating that ~ 25% of the BRN3A^+^ RGC population and axons were lost; these numbers increased slightly (to 30%) by 17 weeks. In sum, these data show that axons are lost early after injury, before RGC somas are lost, and glia are involved in the damage induced by bTBI in the retina.

This study reports a timeline for the loss of RGCs, axons, and cells of the retinal GCC (Fig. 3e), as well as in vivo changes in the retinal ganglion cell layer and an analysis of the retinal transcriptome in a mouse model of bTBI. Interestingly, much of the damage occurred between 1 day and 1 week, and both microglia and macroglia seemed to be active during this period. By 5 weeks most of the axons and RGCs had been lost and macroglia and microglia activity appeared to have subsided. Nevertheless, neurodegeneration continued, mostly in the optic nerve, where the number of degenerating myelinated axons remained high. By 17 weeks, in contrast, there was no significant difference in the percentage of degenerating myelinated axons in bTBI- and sham-treated optic nerves. Thus, whereas vision defects in humans exposed to mild-bTBI are thought to take a long time to develop, our current data show that rapid biological changes can cause irreversible loss of neurons. How these changes correlate to higher order visual processing, and whether they are exacerbated by advancing age, are important questions to answer in future work.

Several studies using the same chamber have consistently found RGC damage^19–21,34^, but most have only examined a limited number of timepoints, with the earliest timepoint previously documented to have RGC loss being 5 weeks post-injury^20^. Some studies using the same chamber have used different PSI pressures, or varied the number of exposures, blurring the ability to make comparisons^22,24^. Previous work in our model, reported a reduction of 14.9% in total density of cells in the RGC layer at 16 weeks, when both eyes were assessed^19^ for a single exposure for the injured mice compared to sham (quantified in whole-mount retinas). Meanwhile, the density of BRN3A^+^ RGCs at 16 weeks after injury^20^ decreased by 27.3% when the eye exposed directly to the overpressure wave was assessed. For multiple exposures with a 1-week inter-exposure interval, the density of BRN3A^+^ RGCs was reduced by 21.96%, and for a 1-h inter-exposure interval it was reduced 14.4%, in the retinas of eyes exposed to blast compared to their sham treated controls^20^ (quantified in whole-mount retinas). Thus, when accounting for the differences in procedures of the past experiments, the end-stage of damage currently reported in this study is similar in scale to previous work.

Damage to RGC and optic nerve have not been consistently studied in other mouse models of blast injury. In some models, only RGC loss was reported^34,35^, while some of the mouse models of blast injury are characterized by traumatic optic neuropathy and can be used to model indirect traumatic optic neuropathy in the context of head trauma^36–38^. Other models of indirect trauma-induced injury of the optic nerve use external ultrasound energy instead of an air blast^39,40^. For example, loss of RGCs was reported in a mouse model using a shock tube generating a pressure of 269 kPa (39 PSI) to expose the top of the head to the pressure wave, the RGC layer had 33.4% fewer cells at 24 days after exposure^34^ (quantified by H&E staining of retinal sections). Damage to the retina and optic nerve, including a decrease in the number of axons, were reported in a model of ocular blast injury^35,41–44^. For example, in the ocular blast (50 PSI) injury model, RGC loss was observed by quantifying an ~ 16% decrease in Thy1-CFP^+^ RGCs^35^ at 6 weeks after injury (quantified in whole-mount retinas). A recent study using the ocular blast injury model with multiple exposures to 15 PSI blasts quantified simultaneously the loss cells from the RGC layer and of axons at multiple time^45^. The authors reported a significant loss of cells in the ganglion cell layer starting two days after injuries as quantified using a nuclear stain DAPI, but no further cell loss up to 30 days post-injury. The loss of myelinated axons determined by PPD staining, was also significant at 2 days post injury and it continued up to 30 days after, with the peak of degeneration observed at 2 weeks^45^. Similar to what we observed in our model, the loss of cells and axons starts early after injury in the mouse model of ocular blast injury, and the loss of axons seems to take longer to complete than the loss of cell soma.

A comparison of our mild bTBI model with other mouse models characterized by RGC dysfunction and loss, such as optic nerve crush (ONC), shows similarities with respect to both the molecular and structural changes that are triggered^46–50^. ONC models direct optic nerve injury and is used for the study of traumatic optic neuropathy and glaucoma. For example, quantification of RGCs and axons after ONC showed that: at 1 day post ONC, a small but significant number of axons, but not RGCs, were lost; at 3 days, RGC loss was significant but still lagged behind the loss of axons; and at 7 days the losses of RGC soma and axons were similar^47^. That study did not report loss of cells from the ganglion cell layer, but analysis by SD-OCT showed that at 7 days post ONC the thickness of the GCC did not differ significantly in the injured vs sham-treated eyes^47^. Our observations in the bTBI model are similar to those for the ONC model with respect to both the histology and the SD-OCT analysis at the 1-week timepoint.

Another similarity between the outcomes of our mild bTBI model and ONC models is that the susceptibility of RGCs to injury varies by subtype, of which 40 have been identified based on functional and transcriptomic characteristics^49,51^. In both ONC and the bTBI model, cells expressing markers for intrinsically photosensitive RGCs (*Opn4*) or alpha-RGCs (*Kcng4*) seem to be more resilient to injury^49,50,52,53^ (Figs. 6b, 7e,f).

Analysis of the retinal transcriptome was also consistent with our observation that most of the damage induced by bTBI in our model takes place early. We chose 1-week post bTBI as the first timepoint because the RGCs were damaged and axons were degenerating, and 5 weeks post bTBI because by then most of the RGCs were already lost, but some axons were still in the process of degenerating. Strikingly, the differences in the distributions of the upregulated and downregulated transcripts at the two timepoints were large. At the 1-week timepoint, expression of 58.8% of the transcripts was increased in the bTBI vs sham retinas (197 out of 335), whereas at 5 weeks after injury the expression of only 2.3% of the transcripts was increased (4 out of 175). The transcripts that were downregulated at both timepoints (96 out of 137 at 1 week, and 171 out of 175 at 5 weeks, post injury; Supplementary Table S5 online) belong to similar pathways that are related to RGCs and to synaptic processes. This analysis suggests that the processes that occur at 1 week, including the activation of microglia and macroglia, are completed by 5 weeks after injury. In combination with the structural analysis, the evaluation of the retinal transcriptome indicates that in the mouse model of bTBI, the time-window for the prevention of the majority of cell and axon loss, and for the glial activation, is the first week after injury. Our examination of the retinal transcriptome also enabled us to determine whether retinal cells other than RGCs were lost. The analysis of the transcripts encoding cell type-specific markers indicated that only the RGC population was reduced after bTBI in our model, consistent with findings from earlier studies^19–21^.

Activation of glia within the retina is thought to play an important role in retinal injury resulting from damage to the optic nerve^54^, and the transcriptome analysis of the injured retinas in our model of bTBI along with immunohistochemical staining indicate that glia play a similar role in this setting. Furthermore, involvement of microglia, astrocytes, and Müller glia (the main macroglia of the neural retina) in the development of retinal damage after exposure to blast is consistent with other reports in the literature. Rodent studies of both blast-induced trauma models and indirect traumatic neuropathy models have reported changes in the numbers of astrocytes and microglia present in the brain^55^, retina, and optic nerve^35,42,56–58^. The numbers of microglia, astrocytes, and Müller cells were also elevated in retinas of mice exposed to multiple blasts in the same enclosed chamber that we used, at 1-week post injury^22^.

GFAP has been proposed as a biomarker in humans for TBI^59–61^, including bTBI^62–64^. Activation of astrocytes and Müller glia in the retinas of mice exposed to blast is indicated by an increase in the markers *Vim* (Müller glia), *Gfap* and *Nes* (Müller glia and astrocytes)^65,66^. Müller glia are considered the main regulators of neuronal signaling in the retina and they are thought to be either beneficial (provide neuroprotection) or destructive, depending on the situation^65,67,68^. The observed increases in levels of transcripts that are characteristic of activated macroglia and in GFAP staining at 1-week post injury (when RGC damage is developing) and the normalization of the transcript levels at 5 weeks (after most RGC damage has occurred) are strong indications that macroglia play a role in the retinal damage that follows bTBI and that this should be further explored.

Besides the involvement of macroglia, the analysis of the retinal transcriptome also suggested that microglia have a role in the processes that take place after bTBI in the retina. The increase in the number of IBA1^+^ cells observed in the whole mount retinas at 1-week post-injury (Fig. 7) confirmed that hypothesis. Microglia can protect against transient pathophysiological insults and are thought to help maintain the structure and function of synapses, yet in diseased states they can contribute to neurodegeneration^69,70^. In mouse models of glaucoma, microglia: are activated before RGCs are lost; can express proinflammatory cytokines and molecules from the complement pathway capable of opsonizing synapses; and can phagocytose RGCs^69,70^. In our bTBI model several members of the complement pathway were expressed at higher levels, i.e., *C1qa*, *C1qb* and *C1qc*, *C1ra* and *C4b* (Supplementary Table S3 online). The complement system is a component of the innate immune response, and it participates in inflammatory responses. It has been also been reported to be dysregulated in models of diseases that involve the death of RGCs, for example glaucoma^71–75^. Elevations in levels of components of the complement system were also detected in the cerebrospinal fluid of human TBI patients^76,77^ and the complement system is thought to play a role in TBI^64,78–81^. The C1 proteins can participate in the clearance of pathogens and dead cells, and the heterotrimeric C1q molecule binds to damaged synapses, marking them for opsonization and phagocytosis. Phagocytosis is one of the biological processes for which blast exposed retinas are enriched at 1-week post bTBI (Fig. 5a), and synaptic processes are affected at both 1 week and 5 weeks (Supplementary Fig. S3 online) after injury. These findings are consistent with microglia participating in the removal of damaged RGCs, as well as of vulnerable synapses in the retinas of mice exposed to blast. Based on our results, in this model glia may initiate the cell death or respond to the insult in the retina after bTBI, but seem to not be involved in a chronic response.

Dysfunction in synaptic transmission and reduction in the expression of synaptic proteins have been reported in other models of blast-induced TBI in the brain^82–85^, the retina^86^ and the auditory system^87^. Nevertheless, information about the molecular changes that occur at synapses after exposure to blast is scant. In our model, synaptic processes and the regulation of membrane potential were affected (Fig. 5b,6e). For example, the transcripts of several components of the SNARE complex, which is involved in the vesicle docking and neurotransmitter release, were expressed at lower levels (Supplementary Tables S1 and S2 online). Other transcripts that are expressed at lower levels in the synapses of our model are *Shank1* and *Shank3*, scaffolding proteins present at excitatory synapses^88^ that have been implicated in neuropsychiatric disorders^89,90^. This is similar to findings from other models of TBI such as fluid percussion^91^, and controlled cortical impact^92^.

Our study has several caveats. One is that the loss of axons in the optic nerve was determined at a single discrete location rather than along the whole length of this structure. Analysis of the whole optic nerve would enable an evaluation of the directionality of the damage, which will be important to establishing the mechanism underlying optic-nerve damage in this context. The second caveat is that only myelinated axons are quantified by PPD staining. Notably, the proportion of unmyelinated axons in mature mice has been estimated to be between 9 and 12% depending on the age of the mouse^93^, therefore by analyzing the myelinated axons we expect to be evaluating the majority of RGC axons. Nevertheless, it will be important to determine whether unmyelinated axons are also lost. In future studies we will aim to address both of these caveats.

A third caveat of this study regards the use of BRN3A as an immunohistochemical marker for RGCs, because previous studies suggested that it does not stain all RGCs. Recent studies that analyzed RGC transcriptome at single cell level showed that *Pou4f1* is expressed at some level in all RGC subtypes^49,94^. Immunohistochemical studies suggest that in mouse retinas the BRN3A antibody binds to 80–90% of RGCs^27,95–97^ and that by using this antibody not all types of RGCs are visualized. It has also been suggested that BRN3A is a flawed phenotypic marker for RGCs because the decrease in BRN3A expression as determined by western blot is significant, while the loss in the number of cells at the same time-point is not^95,97^. Nevertheless, multiple studies showed that BRN3A counts are accurate after optic nerve crush when compared to Fluorogold counts^48,50^. To account for these issues and to verify our methodology, in a different group of mice we performed a simultaneous staining with two RGC markers, RBPMS and BRN3A, for the 1-week time-point after bTBI (Supplementary Fig. S2 online). The quantification showed comparable loss of cells expressing the RGC markers, similar to what we observed at transcript level for *Rbpms* and *Pou4f1*. Given our results, and also that BRN3A is a nuclear protein and a larger number of BRN3A^+^ RGCs can be sampled and counted with semi-automated methods, we continued using BRN3A as an RGC marker. Also, to account for the possible lower BRN3A expression in dysfunctional RGCs, the counting protocol for the BRN3A^+^ cells did not exclude any cells based on intensity of staining, so even if they expressed less BRN3A they were included in the count.

We propose that bTBI leads to axon damage and loss before loss of the RGC soma. This is based on our observations that at 1-week post injury, the numbers of BRN3A^+^ and RBPMS^+^ RGCs in the retina and the number of axons in the optic nerve decrease, while there is a lack of an effect on the overall number of cells in the RGC layer. These findings indicate that the BRN3A^+^ and RBPMS^+^ RGCs are still present (as detected by H&E staining) but dysfunctional, and are not stained by the BRN3A and RBPMS antibodies. Although some of the cells detected by H&E could be microglia, our immunostaining results showed that the increase the number of these cells (IB1A^+^) was small (~ 60 cells/mm^2^) while the decrease in the number of BRN3A^+^ RGCs was larger (~ 550 cells/mm^2^). Thus, a majority of the extra cells stained with H&E were RGCs that no longer expressed BRN3A.

This study refines the timeline for the damage and loss of RGCs and axons in a mouse model of bTBI, and based on our results we propose that axonopathy precedes cell death during the development of the retinal damage associated with bTBI. Analysis of the retinal transcriptome and immunohistochemical staining provide information on the underlying molecular changes, suggesting that: both microglia and macroglia are involved; RGC subtypes respond differently to blast exposure; and only RGCs are affected in our model of bTBI. Future studies will build on these findings and further explore the molecular and cellular changes induced by exposure to blast in the visual system.

## Experimental methods

Animal studies were approved by Iowa City VA Health Care System Institutional Animal Care and Use Committee, in compliance with the PHS Policy on Humane Care and Use of Laboratory Animals, and conducted in accordance with The Association for Research in Vision and Ophthalmology Statement for the Use of Animals in Ophthalmic and Vision Research. This study was carried out in compliance with the ARRIVE guidelines. Adult same sex litter mates were housed together in individually ventilated cages (20 × 35 ×14 cm) up to five per cage based on weight. All the mice were maintained on a regular diurnal lighting cycle (12 light: 12 dark) with ad libitum access to food (7012 Harlan Teklad or other approved diet) and water. Temperature and humidity were maintained at species appropriate levels. A mixture of paper and wood chip was used for bedding. Enrichment material was provided daily, consisting of plastic, nylon and wood chews (Bio-Serv), compressed paper for nest building, plastic huts and sterile food treats hidden in their cages (Bio-Serv). Mice were housed in a modern, purpose-built vivarium under specific pathogen free conditions at the Veterans Affairs Health Care Center in Iowa City, Iowa which is accredited by AAALAC (Association for Assessment and Accreditation of Laboratory Animal Care International). Programs for effective sanitation, vermin detection and sentinel health surveillance are in place and the facility is supported by a laboratory animal veterinarian.

Male C57BL/6J mice (The Jackson Laboratory) were subjected to blast injury at 13–17 weeks of age. Mice not exposed to blast were treated in a similar manner, but not exposed to the blast wave, and they will be referred to as sham treated. All analyses were performed in the eye exposed directly to the blast wave. A total of 250 mice were used for the experiments described in this manuscript. Retinal BRN3A labeling and optic nerve PPD staining were performed in tissues from the same mice, in 4 cohorts of mice that were euthanized at 1 day (20 mice), 1 week (20 mice), 5 weeks (20 mice), and 17 weeks (20 mice) post injury. The SD-OCT, H&E staining of the retina and measurements of retinal area were performed in 3 other cohorts of mice euthanized at 1 week (30 mice), 5 weeks (20 mice), and 16 weeks (20 mice) post-injury. RNA sequencing was performed at 1 week (12 mice) and 5 weeks (12 mice) and quantitative reverse transcription PCR at 1 week (10 mice) and 5 weeks (10 mice). Immunostaining was performed at 1 week for IBA1 (17 mice), RBPMS (20 mice), GFAP (10 mice) and at 16 weeks for OPN4 (9 mice).

### Induction of blast injury

Mice were exposed to an overpressure wave of 20 PSI generated in an enclosed dual compartment chamber, as described previously^19,21,98^, using a Mylar membrane (Mylar A, 0.00142 gauge; Country Plastics, Ames, IA). A mouse was anesthetized with a combination of ketamine (45–50 mg/kg body weight, intraperitoneal, IP) and xylazine (8–9 mg/kg body weight, IP), and then placed in a padded protective restraint^19,21^ in the unpressurized side of the chamber. Only the left side of the unrestrained head was exposed directly to the overpressure wave. To prevent hypothermia, the mice were kept on a heating pad after injection of the anesthetic and during recovery from general anesthesia, but not during exposure to blast. Ocular complications related to anesthesia^99^ were minimized by reversing the effects of xylazine with yohimbine hydrochloride (1.5 mg/kg, IP). Also, immediately after recovery from the procedure, buprenorphine was administered as an analgesic (0.1 mL/20 g body weight, 0.003 mg/mL).

### Spectral domain optical coherence tomography (SD-OCT)

A Spectralis SD-OCT (Heidelberg Engineering, Vista, CA) imaging system coupled with a 25D lens for mouse ocular imaging (Heidelberg Engineering, Vista, CA) was used. Mice anesthetized with a ketamine (55–60 mg/kg, IP) and xylazine (10–10.5 mg/kg, IP) cocktail were kept on a heating pad to maintain body temperature. Pupils were dilated with 1% tropicamide ophthalmic solution. The cornea was moisturized with balanced saline before and during recording, and after with 0.3% hypromellose. Volume scans with a pattern size of 20^o^x25^o^ and 61B lines were recorded to quantify the thickness of the GCC (layers containing RGC bodies + axons + dendrites). Scans were analyzed by an individual blinded to the treatment of the mouse, in the superior retina (see Supplementary Fig. S3 online), and blood vessels were excluded from the calculation of GCC thickness.

### Tissue collection, histology and immunohistochemistry

Mice were euthanized with CO_2_ and transcardially perfused with Dubelcco’s phosphate buffered saline (DPBS; Gibco), followed by 4% paraformaldehyde solution in PBS. Whole eyes were enucleated, after which the posterior cups were dissected and fixed for 4 h in 4% PFA in PBS at 4 °C. The primary and secondary antibodies used for immunofluorescence staining are listed in Supplementary Table S6 online, along with the dilution, and incubation time and temperature at which they were used.

### Staining of retinas

For both immunohistochemical labeling of BRN3A and H&E staining of whole-mount retinas, previously published protocols were used^20,100,101^. The initial steps for both of these procedures were the same. Briefly, the posterior cups of the eyes were incubated in a 0.3% Triton-X100 solution in 1 × DPBS overnight at 37 °C (PBST), washed with DPBS. They were then incubated in a solution of 3% hydrogen peroxide and 1% monobasic sodium phosphate at room temperature for 20 min, after which the retinas were dissected and further incubated in 3% hydrogen peroxide and 1% monobasic sodium phosphate for a total of 3 h, followed by washes with DPBS.

For H&E staining, retinas were mounted on Fisherbrand Superfrost Plus Microscope Slides, and either air-dried or mounted with AquaMount overnight. Aquamount was removed by incubation in DPBS, just before the H&E staining. The air-dried retinas were stained as soon as they were dry. Briefly, after incubation in water for 1 min, the slides were stained for 1–1.5 min with Harris Hematoxylin, followed by 10 dips in two different water containers, 3 dips in acid alcohol (0.185% HCl in 70% ethanol), 10 dips in water, 1 min in Bluing reagent, 1 dip in water, 1 min in 80% ethanol, 1–2 dips in Eosin-Y alcoholic, 10 dips in two separate 95% ethanol containers, 10 dips in two separate 100% ethanol containers, and 10 dips in two separate xylene containers. Samples were them mounted in Surgipath MM24 Mounting Medium.

BRN3A immunostaining of RGCs was performed as described earlier^20,100^. The information on the antibodies and incubation conditions are included in Supplementary Table S6 online. Briefly, after washes with DPBS, retinas were incubated in PBST for 15 min at -80 °C (for permeabilization) and blocked in a solution of 2% normal donkey serum in PBST overnight. Retinas were incubated with primary anti-BRN3A antibody in 2% normal donkey serum, 1% Triton-X 100, and 1% dimethylsulfoxide for 48 h at 4 °C for, and with secondary antibody, Alexa Fluor 488 donkey anti-goat, for 4 h at room temperature. For double staining (anti-BRN3A with anti-RBPMS, anti-IBA1, anti-GFAP, or anti-OPN4), after incubation with anti-BRN3A, as described above, the second primary antibody was added over-night. The incubation with the secondary antibodies was also sequential. Cell nuclei were stained with TO-PRO-3 Iodide (1:1000) in DPBS by incubating for 20-min. Retinas were flat-mounted on Fisherbrand Superfrost Plus Microscope Slides, using ProLong Diamond Antifade Mountant, and cover-slipped.

### Quantification of BRN3A^+^ RGCs from whole-mount retinas

For each retina, 12 confocal (Zeiss LSM 710, Zeiss) images were collected (1024 × 1024 pixels, 0.18 mm^2^ image area) from non-overlapping fields at each of three zones of eccentricity (4 central, 4 mid-central, 4 peripheral; see Supplementary Fig. S3 online), and Z-stacks of 3–5 images were collected with a 20X lens. ImageJ was used for quantification of the labeled cells. The first step was to Z-project at maximum intensity, followed by use of the Subtract Background tool with the rolling-ball radius set to 35 pixels, followed by use of the Smooth tool. Huang thresholding was used for conversion to binary images, that were further processed using the Open, Watershed, and Fill Holes functions. For counting of the cells using the Analyze Particles function, the particle size was set to 20–150 μm^2^ and circularity to 0–1. Images were taken and analyzed by individuals blinded to the treatment of the mouse.

### Quantification of RBPMS^+^ and BRN3A^+^ cells from whole-mount retinas

Images were taken in the same manner as for the BRN3A-only stained retinas, but only 8 images (4 mid-peripheral and 4 peripheral) were used for manual counting of stained cells. For counting, the images were processed by using the Z-project function at maximum intensity in ImageJ. The next steps were: CLAHE with the settings block size 50, bins 256, max slope 5 and for mask None; Enhance contrast with saturated pixels 10% and normalization; Subtract Background tool with rolling ball radius 20 pixels; Gaussian blur filter with Sigma (radius) of 0.5 µm; and Enhance contrast with saturated pixels 10% and normalization. The RBPMS^+^ and BRN3A^+^ cells were counted manually on the same images. Images were taken and analyzed by individuals blinded to the treatment of the mouse.

### Quantification of IBA1^+^ and OPN4^+^ cells from whole-mount retinas

Images were taken in the same manner as for the BRN3A-only stained retinas, but in layers with BRN3A^+^ cells Z-stacks of 6–10 images for IBA1 and of 3–5 for OPN4, were collected to include all the positive cells. For counting, the images were Z-projected at maximum intensity in ImageJ, and the IBA1^+^ or OPN4^+^ cells were counted manually. Images were taken and analyzed by individuals blinded to the treatment of the mouse.

### Quantification of area stained by GFAP in whole-mount retinas

Images were taken in the same manner as for the BRN3A-only stained retinas, but in layers with BRN3A^+^ cells, Z-stacks of 4–6 images were collected to include all the stained area. The images were Z-projected at maximum intensity in ImageJ and converted to 8-bit type. Default thresholding was used for conversion to binary images, and the stained fraction area was determined.

### Quantification of H&E-stained cells in the ganglion cell layer

Twenty-four retinal images (Supplementary Fig. S3 online) were collected (BX52; Olympus, Tokyo, Japan) and analyzed using ImageJ and the plugin RetFM-J as previously described^100^. Images were taken and analyzed by individuals blinded to the treatment of the mouse.

### Measurement of total retinal area

H&E-stained whole-mount retinas were scanned at 20 × magnification (ScanScope CS, Aperio, Vista, CA). The total area of each retina was determined using the “Color Deconvolution v.9” algorithm to measure the stained area, and the optic nerve was not included.

### Optic-nerve staining with PPD

Optic nerves were fixed in the skull for 24 h in 4% PFA in DPBS at 4 °C and then processed as described previously^20,102–104^. Briefly, nerves were dissected and fixed overnight in 2.5% glutaraldehyde and 2% PFA in 0.1 M sodium cacodylate buffer pH 7.4, rinsed in 0.1 M sodium cacodylate buffer, and post-fixed with 1% osmium tetroxide for 1 h. A series of 40-min incubations in graded acetone was followed by infiltration overnight at 4 °C with 33%, 66%, and 100% resin (Eponate 12; Ted Pella, Redding, CA, USA) diluted in propylene oxide, and by embedding in 100% resin. Staining with 1% PPD was performed on 1-μm cross sections, followed by mounting with Permount.

### Quantification of axons from PPD-stained optic nerves

Axons were quantified as described earlier^102^. Images were taken at total magnifications of × 100 and × 1000 (BX52; Olympus, Tokyo, Japan). The optic-nerve area was determined by manually tracing and measurement of the cross-sectional area of whole nerves from 100 × images using Image J. A counting frame equivalent to 1% of the total area of the optic nerve was placed randomly within each of 10 images collected at 1000 × magnification. Myelinated healthy and degenerating axons were marked and counted inside the counting frame, and approximately 10% of the optic nerve area from each mouse was analyzed in this manner. PPD stains myelin and healthy axons have clear axoplasm, surrounded by darkly stained myelin and the degenerating axons stain grey/black, including their axoplasm^105^. The total number of axons was extrapolated based on the total area of the optic nerve. Images were collected and analyzed by an individual blinded to the treatment of all mice.

### Tissue collection and RNA extraction

Mice were euthanized by cervical dislocation, and eyes enucleated and immersed in RNA*later* solution. Retinas were dissected rapidly in RNA*later*, and stored in RNA*later* solution. For the 5-week timepoint, total RNA was extracted using the RNeasy Mini Kit (Qiagen Gmbh, Hilden, Germany) and for the 1-week timepoint it was extracted using the RNeasy Plus Mini Kit (Qiagen Gmbh, Hilden, Germany), following the manufacturer’s instructions. Lysis was performed with the TissueLyser LT (Qiagen Gmbh, Hilden, Germany) bead mill, using one steel ball at 50 Hz for 5 min on ice. Samples were analyzed using a NanoDrop 2000 Spectrophotometer (ThermoFischer Scientific), and stored at − 80 °C. Evaluation of RNA quality, the creation of libraries, and RNA sequencing were performed at the Iowa Institute of Human Genetics: Genomics Division, University of Iowa Carver College of Medicine. The RNA integrity number (RIN) values for the samples were between 8.4 and 9.6, and were determined using a Nano RNA chip with Agilent 2100 Bioanalyzer (Agilent Technologies, Inc, Waldbronn, Germany).

Transcription profiling by RNA-Seq was performed using manufacturer-recommended protocols. Initially, 500 ng of DNase I-treated total RNA was used to enrich for polyA-containing transcripts, using oligo(dT) primers bound to beads. The enriched RNA pool was fragmented and converted to cDNAs, which were ligated to sequencing adaptors containing indexe sequences using the Illumina TruSeq stranded mRNA sample preparation kit (Cat. #RS-122-2101, Illumina, Inc., San Diego, CA). The molar concentrations of the indexed libraries were measured using the 2100 Agilent Bioanalyzer (Agilent Technologies, Santa Clara, CA) and combined equally into pools for sequencing. The concentrations of the pools were measured using the Illumina Library Quantification Kit (KAPA Biosystems, Wilmington, MA) and the pools were sequenced on the Illumina HiSeq 4000 genome sequencer using 75 bp paired-end SBS chemistry.

### RNA-Seq analysis

The reads from the RNA-Seq experiment were mapped to the reference mouse genome (GRCm38) using STAR (version 020201^106^). Expression was quantified using the featureCounts function of Rsubread^107^. For each timepoint, differential expression analysis was performed on retinas from sham-treated (n = 6) and injured (n = 6) mice using edgeR^108^. Overrepresentation analysis was performed on the set of differentially expressed genes at each time-point (FDR < 0.005) using the WebGestalt 2019^109^ and the Biological Process ontology from the Gene Ontology knowledgebase^110^.

#### Quantitative reverse transcription PCR (RT-qPCR)

Retinas were collected from new cohorts of mice at 1-week and 5-weeks after injury. For each group (injured or sham-treated) and timepoint, five mice were analyzed. Lysis was performed as for the RNA-Seq experiment, and the RNeasy Plus Mini Kit (Qiagen Gmbh, Hilden, German) was used,with an added step of DNA digestion on the column (RNAse-free DNAse kit Qiagen Gmbh, Hilden, Germany). A NanoDrop 2000 Spectrophotometer (ThermoFischer Scientific) was used for RNA quantitation. Reverse transcription was performed using the High-Capacity cDNA Reverse Transcription kit (Applied Biosystems, Thermo Fisher Scientific, Waltham, Massachusetts, USA) following the manufacturer’s instructions. Predesigned TaqMan Gene Expression Assays (Supplementary Table S7 online) (Applied Biosystems, Thermo Fisher Scientific, Waltham, Massachusetts, USA) were used to determine levels of the mRNAs of interest. These were then normalized to levels of the β-actin (*Actb*) or glyceraldehyde 3-phosphate dehydrogenase (*Gapdh*) mRNA, as determined using a real-time PCR detection system (C1000 Thermal Cycler; Bio-Rad Laboratories, Hercules, CA, USA). The composition of the reaction mix was 1 × TaqMan Universal PCR Master Mix (Applied Biosystems, Thermo Fisher Scientific, Waltham, Massachusetts, USA), 1 × TaqMan Gene Expression Assay for the target mRNA, 0.75 × TaqMan Gene Expression Assay (see Supplementary Table S7 online) for *β-actin* or *Gapdh* and 0.512 ng/uL RNA for *Nefl* and *Tubb3*; and 2.72 ng/uL for *GFAP*, *C1qa*, *Spp1* and *Pvalb*. The PCR conditions were: 50 °C for 2 min, 95 °C for 10 min, and 40 cycles of (95 °C for 15 s, 60 °C for 1 min). For each sample, three technical replicates were analyzed. For each transcript, the *C*_t_ values for each sample were determined using the Bio-Rad CFX Manager software, and then averaged and normalized to values for *β-actin* or *Gapdh*.

### Statistical analysis

The Prism 8 software (version 8.4.2; GraphPad) was used to generate graphs and perform statistical analysis. Values are expressed as mean ± standard deviation, and data were analyzed using unpaired two tailed *t*-test analysis with Welch’s correction.

## Supplementary information

Supplementary Informations.

Supplementary Table S3.

Supplementary Table S4.

Supplementary Table S5.
