# Supplementary material for: Axonopathy precedes cell death in ocular damage mediated by blast exposure
Source: Sci Rep. 2021 Jun 3;11:11774. doi: 10.1038/s41598-021-90412-2 (PMC8175471; doi:10.1038/s41598-021-90412-2)
Supplement: Supplementary file 1 — Supplementary Informations. [file 41598_2021_90412_MOESM1_ESM.pdf]

Axonopathy Precedes Cell Death in Ocular Damage Mediated by Blast Exposure

Nickolas A. Boehme[1,2], Adam Hedberg-Buenz [1,3], Nicole Tatro[2,4], Michael Bielecki[2], William C. Castonguay[1,3], Todd E. Scheetz[2,4], Michael G. Anderson [1-4], Laura M. Dutca[1,2]

[1]Center for the Prevention and Treatment of Visual Loss, Iowa City VA Healthcare System, Departments of [2]Ophthalmology and Visual Science, [3]Molecular Physiology and Biophysics, [4]Institute for Vision Research, The University of Iowa, Iowa City, IA

Supplemental Table S1. Summary of the mean (±SD) density of BRN3A⁺ RGCs in whole-mount retinas, and total number of axons in the corresponding optic nerves (PPD) for retinas from blast induced-TBI and sham groups at the timepoints indicated.

Timepoint	Treatment	Mean density of BRN3A+ RGCs ±SD (cells/mm^2)	*p*-value	Total number of axons in optic nerves ±SD	*p*-value
1D	Sham	3084±210.2	0.4546	55400±11532	0.6044
	bTBI	3063±227.2		52750±7991	
1W	Sham	3008±288.2	0.021	62386±6329	0.0056
	bTBI	2451±267.2		48117±12854	
5W	Sham	2981±222.1	0.001	65433±15860	0.0373
	bTBI	2130±474		47256±15731	
17W	Sham	2777±317.1	<0.001	52910±13733	0.0197
	bTBI	1933±392		36339±8534	

Supplemental Table S2. Summary of the mean (±SD) GCC thickness, total cell density, and total retinal area for retinas from bTBI and sham groups at the timepoints indicated.

Timepoint	Treatment	GCC thickness[a] ±SD (µm)	*p*-value	Total density[b] ±SD (cells/mm^2)	*p*-value	Total retinal area[b] ±SD (mm^2)	*p*-value
1W	Sham	72.58±2.65	0.1665	8367±546.9	0.6733	14.78±1.335	0.8888
	bTBI	71.2±2.54		8281±451.4		14.7±1.546	
4W/5W	Sham	69.68±1.43	0.0367	8263±621.8	0.0309	16.07±1.379	0.2655
	bTBI	66.7±3.71		7294±1030		15.31±1.451	
16W	Sham	71.94±2.16	0.0006	7852±475.3	0.0046	16.74±1.675	0.5167
	bTBI	67.1±2.66		6806±861.1		17.29±2.005	

[a] GCC thickness was as determined *in vivo* by SD-OCT
[b] Total cell density and total retinal area were determined on whole-mount retinas stained with hematoxylin and eosin

Supplemental Table S3. List of retinal transcripts significantly dysregulated at 1-week post-injury (as excel file) in retinas from blast-induced TBI and sham groups, as determined by RNA sequencing.

Supplemental Table S4. List of retinal transcripts significantly dysregulated at 5 weeks post-injury (as excel file) in retinas from blast-induced TBI and sham groups, as determined by RNA sequencing.

Supplemental Table S5. List of retinal transcripts significantly dysregulated at both 1- and 5-weeks post-injury (as excel file) in retinas from blast-induced TBI and sham groups, as determined by RNA sequencing.

Supplemental Table S6. Primary and secondary antibodies used for immunofluorescence staining.

**Antibody**	**Host**	**Source; Product number**	**Dilution; Incubation time; temperature**
anti-BRN3A	Goat	Santa Cruz Biotechnology; sc8429	1:200; two nights; 4°C
anti-GFAP	Rabbit	Abcam; ab7260	1:500; over night; 4°C
anti-IBA1	Rabbit	Wako; 019-19741	1:500; over night; 4°C
anti-OPN4	Rabbit	Advanced Targeting Systems; AB-N39	1:500; over night; 4°C
Anti-RBPMS	Guinea pig	Milipore Sigma; ABN1376	1:500; five nights; 4°C
anti-goat IgG Alexa Fluor 546	Donkey	ThermoFisher Scientific; A-11056	1:200; 4 hours; room temperature
anti-goat IgG Alexa Fluor 488	Donkey	ThermoFisher Scientific; A-11055	1:200; 4 hours; room temperature
anti-rabbit IgG Alexa Fluor 488	Donkey	ThermoFisher Scientific; A21206	1:200; 1 hour; room temperature
anti-guinea pig IgG Alexa Fluor 594	Donkey	Jackson ImmunoReseach Laboratories; 706-585-148	1:200; over night; 4°C

Supplemental Table S7. TaqMan® Gene Expression Assays used for quantitative reverse transcription PCR validation of RNA sequencing data.

**Gene**	**Assay ID**
Actb	Mm02619580_g1
C1qa	Mm00432142_m1
Gapdh	Mm99999915_g1
Gfap	Mm01253033_m1
Nefl	Mm01315666_m1
Pvalb	Mm00443100_m1
Spp1	Mm00436767_m1
Tubb3	Mm00727586_s1

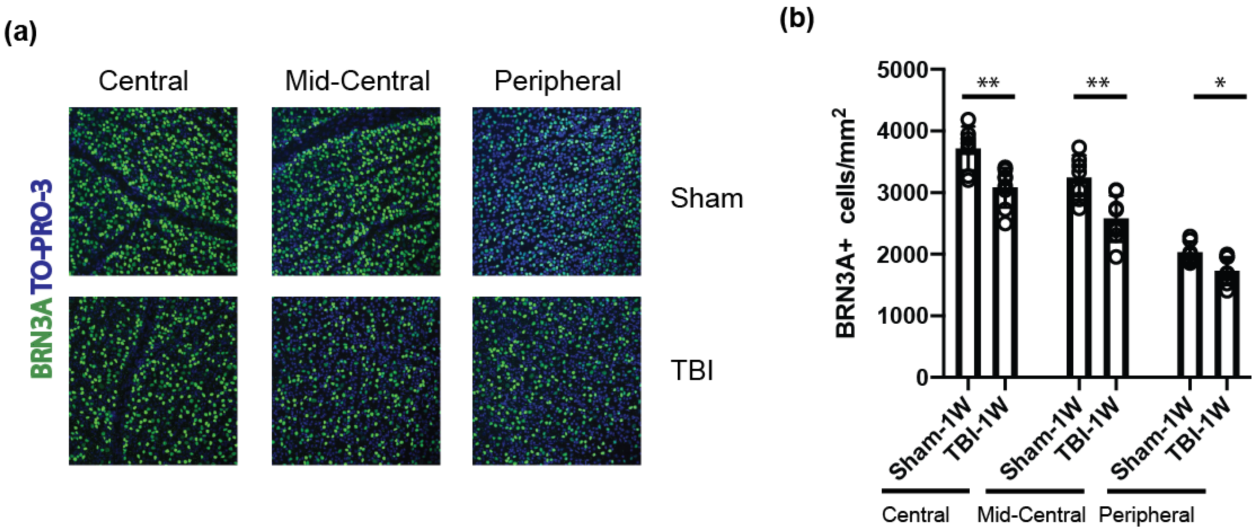

Supplemental Figure S1. Blast-mediated traumatic brain injury (bTBI) was associated with loss of BRN3A^{+} retinal ganglion cells (RGCs) in all retinal areas at 1-week post-injury. (a) Representative images of whole-mount retinas stained with anti-BRN3A antibody (green) and TOPRO3 (blue), from the indicated areas of retinas from bTBI and sham groups of mice. (b) Quantification of BRN3A^{+} RGCs in the indicated areas of retinas at 1 week (1W) after injury, for bTBI and sham mice. Bars represent mean ± SD, and each data point represents a biological sample; unpaired two tailed *t*-test analysis with Welch's correction: *$p < 0.05$, ** $p < 0.01$.

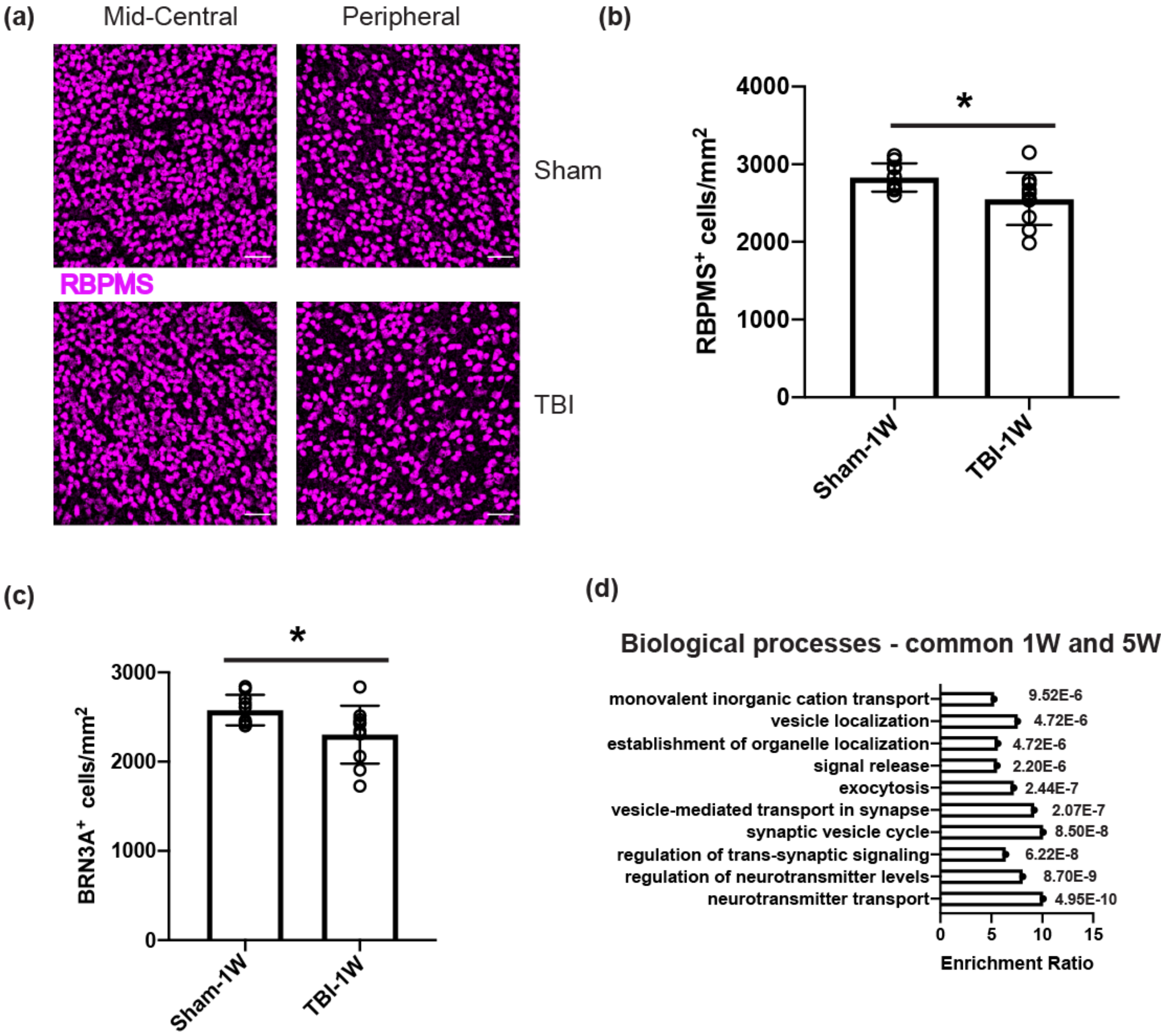

Supplemental Figure S2. Blast-mediated traumatic brain injury (bTBI) was associated with loss of RBPMS$^+$ retinal ganglion cells (RGCs) at 1-week post-injury. (a) Representative images of whole-mount retinas stained with anti-RBPMS antibody (pink) from the indicated areas of retinas from bTBI and sham groups of mice. (b) Quantification of RBPMS$^+$ RGCs from mid-central and peripheral areas of retinas at 1 week (1W) after injury, for bTBI and sham mice. (c) Quantification of BRN3A$^+$ RGCs from mid-central and peripheral areas of retinas at 1W after injury, for bTBI and sham mice. Bars represent mean ± SD, and each data point represents a biological sample; unpaired two tailed *t*-test analysis with Welch's correction: *$p < 0.05$, ** $p < 0.01$. Scale bar represents 50 µm. (d) Transcripts whose expression was significantly dysregulated at 1W and 5 weeks (5W). Transcripts whose expression changed significantly (false

discovery rate (FDR)< 0.005 for TBI compared to sham retinas) analyzed using WebGestalt, by performing an "Over-Representation Analysis" using the gene ontology database for "Biological Process noRedundant" analysis. The false discovery rate (FDR) for each process is indicated.

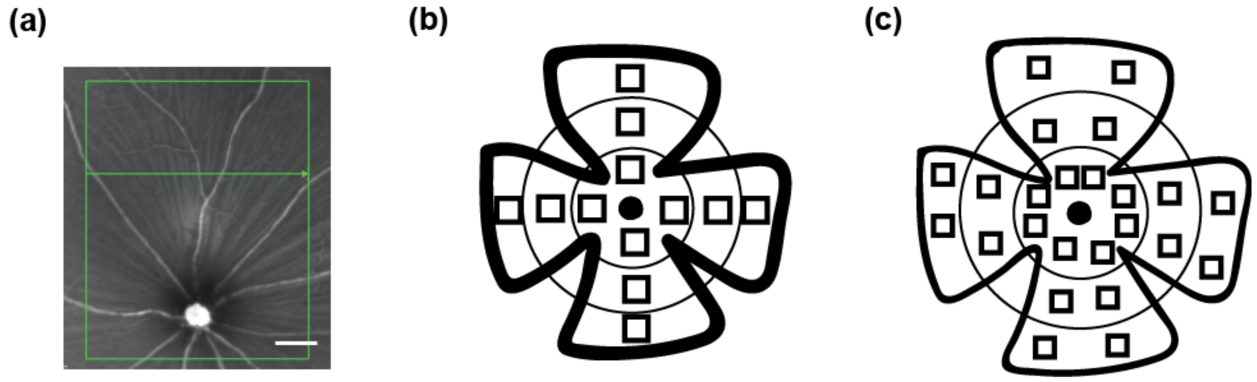

Supplemental Figure S3. (a) Fundus image of the mouse retina, with the location of the layer in which thickness of the ganglion cell complex (GCC) was measured indicated by the long arrow. Scale bar represents 200 μm. (b) Sampling scheme for determining the density in BRN3A$^+$, IBA1$^+$, and OPN4$^+$ cells in whole-mount retinas. (c) Sampling scheme for determining the total cell density in the ganglion cell layer in whole-mount retinas stained with hematoxylin and eosin (H&E).
